# I-125 seeds with chemotherapy for progressive non-small-cell lung cancer after first-line treatment: a meta-analysis

**DOI:** 10.1186/s13019-022-01820-y

**Published:** 2022-04-12

**Authors:** Zhong-Ke Chen, Jing Fan, Fen-Qiang Li, Shi-Yan Zhou, Yuan-Shun Xu

**Affiliations:** 1 Department of Interventional Therapy, Affiliated Hospital of Gansu Medical College, Pingliang, China; 2 Interventional Radiology Department, Lanzhou University First Affiliated Hospital, Lanzhou, China; 3 Radiology Department, Xuzhou Central Hospital, Xuzhou, China

**Keywords:** I-125 seed, Non-small-cell lung cancer, Progressive, Meta-analysis

## Abstract

**Background:**

Continuing therapy for aggressive non-small-cell lung cancer (NSCLC) after first-line treatment (FLT) is challenging. The clinical efficacy of second-line chemotherapy (SLCT) for progressive NSCLC is limited. In this meta-analysis, we aim to evaluate the clinical efficacy of the combination of I-125 seeds brachytherapy (ISB) and SLCT in progressive NSCLC after FLT.

**Methods:**

The PubMed, Embase, Cochrane Library, CNKI, Wanfang, and VIP databases were screened for relevant publications until September 2021. Meta-analyses are conducted by RevMan 5.3 and Stata 12.0.

**Results:**

Our meta-analysis encompassed 6 studies (4 retrospective studies and 2 randomized controlled trials), which included 272 patients that underwent ISB with SLCT (combined group) and 257 patients that received SLCT alone (chemotherapy alone group). The complete response (24.7% vs. 7.0%, *P* < 0.00001), treatment response (65.7% vs. 38.1%, *P* = 0.0002), and disease control (95.2% vs. 80.4%, *P* < 0.00001) rates are markedly elevated for patients receiving combined therapy versus those receiving chemotherapy alone. Moreover, pooled progression-free survival (*P* = 0.0001) and overall survival (*P* < 0.00001) were remarkably extended for patients that received the combination therapy, while no obvious differences were detected in the pooled myelosuppression (39.0% vs. 30.6%, *P* = 0.05) and gastrointestinal response (38.5% vs. 35.9%, *P* = 0.52) rates between 2 groups. Significant heterogeneity was found in the endpoints of the treatment response and progression-free survival.

**Conclusions:**

This meta-analysis demonstrated that ISB could enhance the clinical efficacy of SLCT in patients with progressive NSCLC after FLT without inducing major toxic side effects.

## Introduction

Lung cancer is the leading cause of global cancer death. The most (approximate 75–80%) prominent type of lung cancer is non-small-cell lung cancer (NSCLC) [1–3]. For the patients suffering from inoperable NSCLC, concurrent chemoradiotherapy (CCRT) is regarded as the standard first-line treatment (FLT) [4–6]. When performing the CCRT, traditional external radiotherapy is commonly used [4–6]. However, traditional external radiotherapy is typically associated with radiation- related complications, and the radiological dosing is limited by the distance between the tumor and the surrounding healthy tissue and vital organs [7].

Recently, I-125 seeds brachytherapy (ISB) has been widely used for the treatment of various malignant tumors [7–10]. Compared to the traditional external radiotherapy, ISB has many advantages, including the ease of delivery, direct interaction with the tumor surface, and the sustained application of low-dose radiation to the tumor site over a prolonged duration of time [10]. ISB is often used in combination with systematic chemotherapy or transcatheter arterial chemical infusion in patients with progressive NSCLC [7]. However, a majority of the previous studies focused on the combination of ISB and chemotherapy as the FLT for severe NSCLC patients [7]. However, the number of studies investigating the combination of ISB and chemotherapy treatment for progressive NSCLC after FLT is still limited [11–16]. Therefore, a meta-analysis should be performed to increase the statistical power of the small sample study.

In this meta-analysis, we aimed to evaluate the clinical effectiveness of combined ISB with second-line chemotherapy (SLCT) for treating progressive NSCLC after the FLT.

## Methods

This meta-analysis abided by the Preferred Reporting Items for Systematic reviews and Meta-Analyses (PRISMA) statement [17], and it was registered at INPLASY.COM (No. INPLASY2021100120).

### Study selection

Relevant studies were searched in the following databases: PubMed, Embase, Cochrane Library, CINK, Wanfang, and VIP (until September 2021) using the following search terminologies: ((((((Iodine-125) OR (I125)) OR (125I)) OR (brachytherapy)) AND ((lung cancer) OR (NSCLC))) AND (chemotherapy)) AND ((((recurrent) OR (failure)) OR (second line)) OR (progressive)).

The following articles were included in the current meta-analysis:

- Type of investigation: comparative studies;
- Disease: advanced NSCLC after first-line chemotherapy or CCRT;
- Type of interventions: ISB with SLCT versus SLCT alone;
- Languages: all.

The following articles were excluded from the current meta-analysis:

- studies without any control group;
- case reports;
- meta-analyses and reviews.

### Data extraction

Two independent researchers retrieved the relative data and endpoints, and the disagreements were resolved by a third researcher. The baseline data from each publication included the first author’s name, publication year, countries, types of study design, cancer types, tumor stage, FLT protocol, SLCT protocol, sample size, age, and gender. The outcomes of each study included the following parameters: complete response (CR), treatment response (TR), disease control (DC), myelosuppression, progression-free survival (PFS), and overall survival (OS).

### Quality assessment

Potential bias was evaluated by using the Cochrane risk of bias tool for randomized controlled trials (RCTs) [18]. The items of Cochrane risk of bias tool included the following six domains of bias: performance, attrition, detection, selection, reporting, and other sources of bias.

The overall quality of the non-RCTs were analyzed with the 9-point Newcastle–Ottawa scale (NOS) [19], which classified the studies exhibiting into low, intermediate, or high levels of risk, with the scores of ≥ 7, 4–6, and < 4, respectively. The items of NOS included the non-RCT articles based on nine aspects related to study selection (4 points), comparability (2 points), and exposure (3 points).

### Definitions

Therapeutic efficacy was examined with the Response Evaluation Criteria in Solid Tumors [20]. TR = CR + partial response; DC = CR + partial response + stable disease. PFS was defined as the time from the initial testing to radiographic monitoring of disease progression. OS was calculated as the time from the treatment to death.

CR was described as the complete absence of all target lesions. Partial response was described as a minimum of 30% reduction in the sum of the largest size of the target lesions. Progressive disease referred to a minimum of 20% increase in the sum of the largest size of the target lesions, or formation of new lesions. Stable disease is regarded as not reaching the partial response or progressive disease standard [20].

### Statistical analyses

RevMan v5.3 and Stata v12.0 were employed for this meta-analysis. Dichotomous variables were determined by pooled odds ratios (ORs) with 95% confidence intervals (CIs). Pooled PFS and OS were calculated by hazard ratios (HRs) with 95% CI. Heterogeneity was determined by the *I*^*2*^statistic and Q test. *I*^2^ > 50% was defined as high heterogeneity, and then the random effect model was utilized; otherwise, the fixed effects model was utilized. Heterogeneity sources were evaluated by sensitivity and subgroup analyses. Egger test was used to evaluate publication bias. *P* < 0.05 is the threshold for statistical significance in publication bias.

## Results

### Included studies

**Fig. 1:**
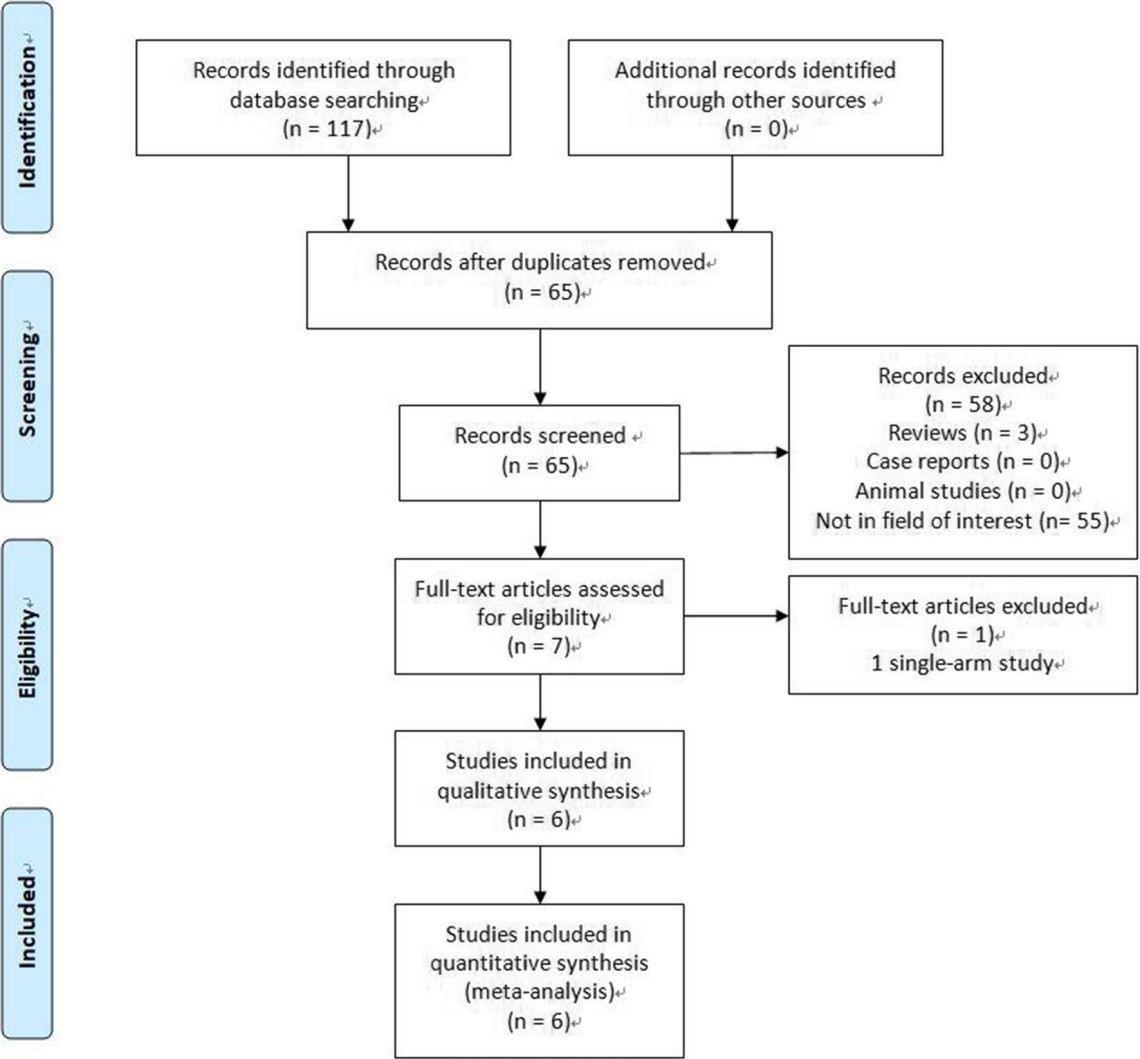
The flowchart of this study

We identified 117 relevant studies by using the research strategy described above. After a full review, only 6 studies fulfilled the selection criteria and they were included into the current meta-analysis (Fig. 1). Among the 6 studies, 272 patients underwent ISB with SLCT (combined group), and 257 patients underwent SLCT alone (chemotherapy alone group, Table 1). All included studies were from China. Two studies were RCTs [11, 14], and 4 studies were retrospective studies [12, 13, 15, 16]. Furthermore, 4 studies used CCRT [11–14] and 2 studies used chemotherapy only as the FLTs [15, 16]. The raw data for CR, PR, SD, and PD are shown in Table 2.

**Table 1:** Characteristics of the included studies

Study/year/country	Study design	Cancer types	Stage	Groups	Sample size	Age (years)	M/F	NOS
Luo/2013/China [11]	RCT	A; SC	III	Chemotherapy alone	17	48–72 for all	18/16 for all	–
				Combined	17			
Xiang/2015/China [12]	Retrospective	A; SC; other	III, IV	Chemotherapy alone	41	56.8	30/11	8
				Combined	37	59.5	25/12	
Xiang/2021/China [13]	Retrospective	A; SC; other	III, IV	Chemotherapy alone	94	55.4	71/23	8
				Combined	116	58.5	81/35	
Yu/2015/China [14]	RCT	A; SC; other	III	Chemotherapy alone	26	62	15/11	–
				Combined	26	62	17/9	
Zhang/2014/China [15]	Retrospective	A; SC; other	III, IV	Chemotherapy alone	35	54	28/7	8
				Combined	34	52	25/9	
Zheng/2020/China [16]	Retrospective	A; SC; other	III, IV	Chemotherapy alone	44	46–70	31/13	8
				Combined	42	45–70	25/17	

*NOS* Newcastle–Ottawa scale, *RCT* randomized controlled trial, *SC* squamous carcinoma; *A* adenocarcinoma, *M* male, *F* female

**Table 2:** Characteristics of the treatments

Study	First-line chemotherapy	Second-line chemotherapy	Groups	CR	PR	SD	PD
Luo [11]	Paclitaxel, Cisplatin*	Docetaxel, Cisplatin	Chemotherapy alone	1	6	6	4
			Combined	7	4	4	2
Xiang [12]	Paclitaxel, Cisplatin, Gemcitabine, Carboplatin*	Docetaxel, Pemetrexed	Chemotherapy alone	1	16	17	7
			Combined	13	14	15	2
Xiang [13]	Not given*	Docetaxel, Pemetrexed	Chemotherapy alone	9	31	38	16
			Combined	21	47	41	7
Yu [14]	Paclitaxel, Cisplatin*	Docetaxel, Pemetrexed	Chemotherapy alone	2	13	8	3
			Combined	10	8	6	2
Zhang [15]	Paclitaxel, Cisplatin, Gemcitabine	Docetaxel, Pemetrexed	Chemotherapy alone	7	17	25	15
			Combined	24	33	10	0
Zheng [16]	Paclitaxel, Cisplatin, Gemcitabine	Docetaxel, Cisplatin	Chemotherapy alone	0	6	29	9
			Combined	2	22	16	2

*CR* complete response, *PR* partial response, *SD* stable disease, *PD* progression disease

*These studies used concurrent chemoradiotherapy as the first-line treatment

### Quality assessments

**Fig. 2:**
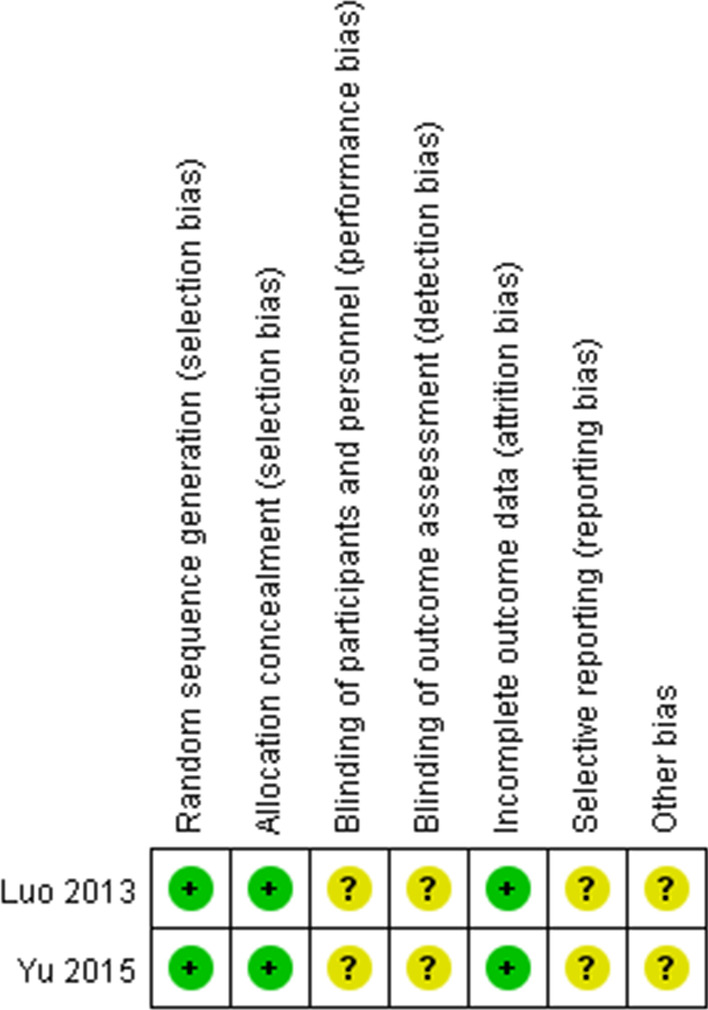
Cochrane’s risk of bias assessment for the included RCTs

The included RCTs had an unclear risk of performance, detection, reporting, and other bias (Fig. 2). The NOS for all the retrospective studies are all 8 (Table1).

### CR rates

**Fig. 3:**
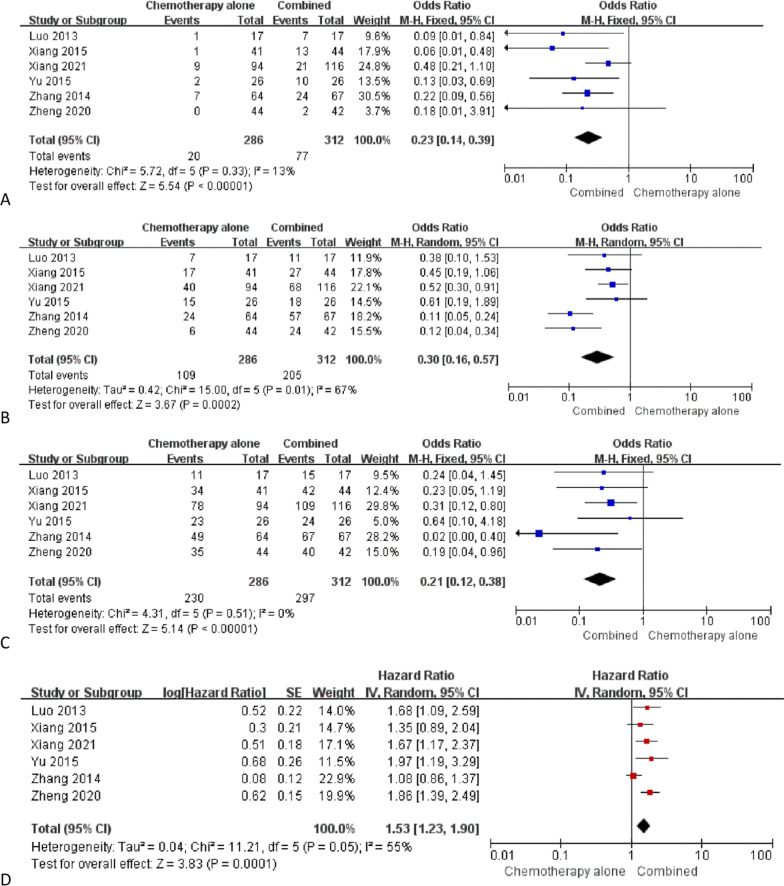

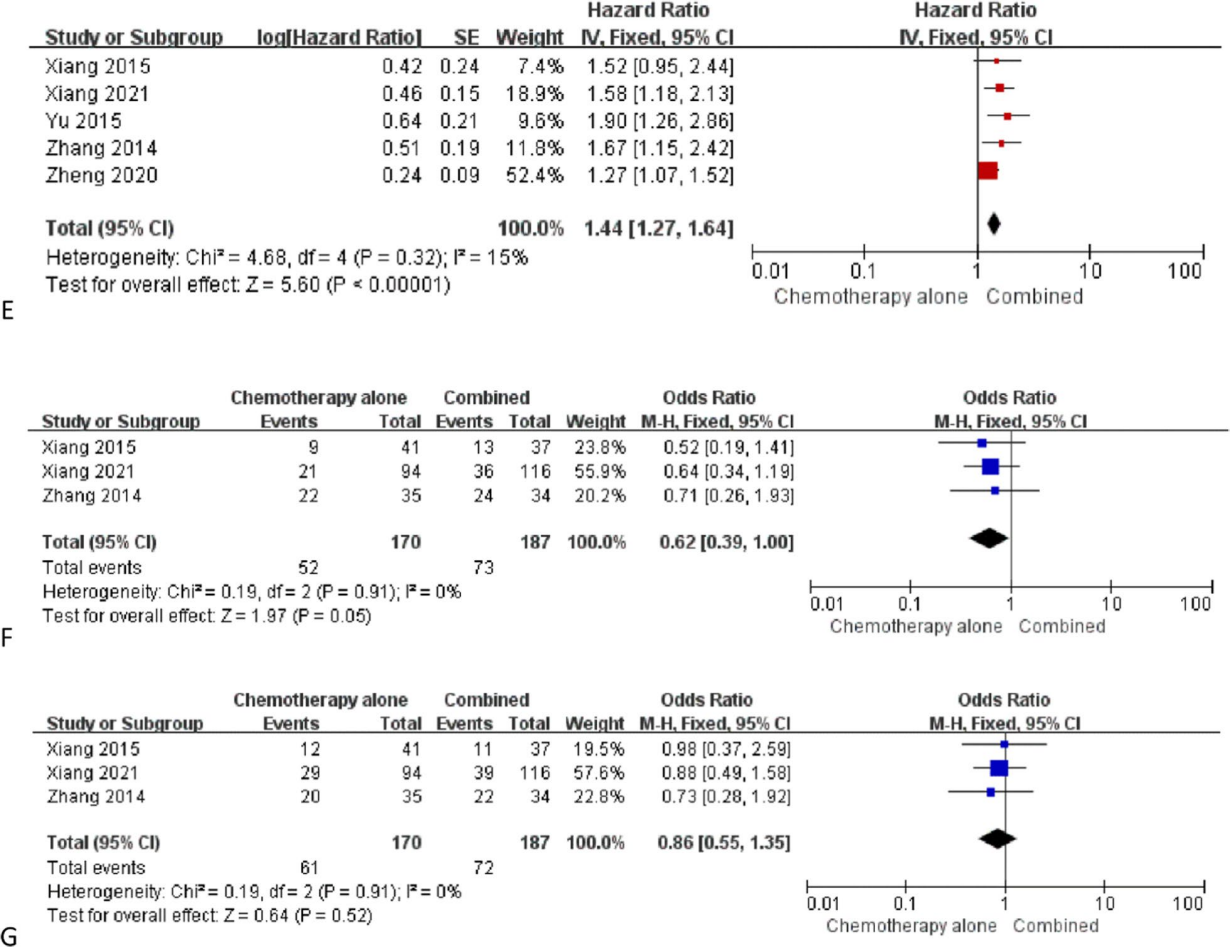
The pooled results of **a** CR rates, **b** TR rates, **c** DC rates, **d** PFS, **e** OS, **f** myelosuppression rates, and **g** gastrointestinal response rates between 2 groups

CR rates could be extracted from all included studies. The pooled CR rate was significantly larger in the combined group than that in the chemotherapy alone group (24.7% vs. 7.0%, *P* < 0.00001, Fig. 3a). The heterogeneity was not significant (*I*^2^ = 13%). The publication bias did not reach statistical significance (Egger test, *P* = 0.503).

### TR rates

TR rates could be extracted from all studies. The pooled TR rate was significantly larger in the combined group than that in the chemotherapy alone group (65.7% vs. 38.1%, *P* = 0.0002, Fig. 3b). The heterogeneity was significant (*I*^2^ = 67%). The publication bias did not reach statistical significance (Egger test, *P* = 0.709).

The sensitivity analysis indicated that the significant heterogeneity disappeared (*I*^2^ = 39%) upon excluding the study from Zhang et al. [15] study. Under this condition, the pooled TR rate was still significantly larger in the combined group than that in the chemotherapy alone group (*P* = 0.0005).

### DC rates

DC rates could be extracted from all studies. The pooled DC rate was significantly higher in the combined group than that in the chemotherapy alone group (95.2% vs. 80.4%, *P* < 0.00001, Fig. 3c). The heterogeneity was not significant (*I*^2^ = 0%). The publication bias did not reach statistical significance (Egger test, *P* = 0.662).

### PFS

The logHR and SE for PFS could be extracted from all studies. The pooled logHR and SE indicated that PFS was significantly longer in the combined group than that in the chemotherapy alone group (*P* = 0.0001, Fig. 3d). The heterogeneity was significant (*I*^2^ = 55%). The publication bias did not reach statistical significance (Egger test, *P* = 0.637).

The sensitivity analysis indicated that the significant heterogeneity disappeared (*I*^2^ = 0%) upon excluding the study from Zhang et al. [15] study. Under this condition, the PFS was still significantly longer in the combined as compared to the chemotherapy alone group (*P* < 0.00001).

### OS

The logHR and SE for OS could be extracted from 5 studies [12–15]. The pooled logHR and SE indicated that OS was significantly longer in the combined group than that in the chemotherapy alone group (*P* < 0.00001, Fig. 3e). The heterogeneity was not significant (*I*^2^ = 15%). The publication bias did not reach statistical significance (Egger test, *P* = 0.682).

### Myelosuppression

Myelosuppression rates could be extracted from 3 studies [12, 13, 15]. The pooled myelosuppression rates were comparable between 2 groups (39.0% vs. 30.6%, *P* = 0.05, Fig. 3f). The heterogeneity was not significant (*I*^2^ = 0%). The publication bias did not reach statistical significance (Egger test, *P* = 0.758).

### Gastrointestinal response

Gastrointestinal response rates could be extracted from 3 studies [12, 13, 15]. The pooled myelosuppression rates were comparable between 2 groups (38.5% vs. 35.9%, *P* = 0.52, Fig. 3g). The heterogeneity was not significant (*I*^2^ = 0%). The publication bias did not reach statistical significance (Egger test, *P* = 0.896).

### Subgroup analyses

Table 3 shows the subgroup analyses based on the study types (RCT or retrospective). According to the RCTs, combination therapy showed better efficacy in terms of the CR rate (*P* = 0.001) and PFS (*P* = 0.0005). Based on the retrospective studies, combination therapy showed better efficacy in the terms of the CR (*P* < 0.00001), TR (*P* = 0.001), DC (*P* < 0.0001) rates, PFS (*P* = 0.009), and OS (*P* < 0.0001). Nevertheless, the significant heterogeneity was found in the endpoints of TR rates (*I*^2^ = 78%) and PFS (*I*^2^ = 67%).

**Table 3:** Meta-analytic results based on the studies regarding of different design types

	Number of studies	OR or HR (95% CI)	Heterogeneity	Favor
Randomized controlled trial				
CR	2	0.11 (0.03, 0.43), *P* = 0.001	*I*^2^ = 0%	Combined
TR	2	0.50 (0.21, 1.21), *P* = 0.13	*I*^2^ = 0%	–
DC	2	0.38 (0.11, 1.36), *P* = 0.14	*I*^2^ = 0%	–
PFS	2	1.80 (1.29, 2.50), *P* = 0.0005	*I*^2^ = 0%	Combined
Retrospective				
CR	4	0.26 (0.15, 0.47), *P* < 0.00001	*I*^2^ = 27%	Combined
TR	4	0.24 (0.10, 0.58), *P* = 0.001	*I*^2^ = 78%	Combined
DC	4	0.18 (0.09, 0.36), *P* < 0.0001	*I*^2^ = 9%	Combined
Myelosuppression	3	0.62 (0.39, 1.00), *P* = 0.05	*I*^2^ = 0%	–
Gastrointestinal response	3	0.86 (0.55, 1.35), *P* = 0.52	*I*^2^ = 0%	–
PFS	4	1.45 (1.10, 1.90), *P* = 0.009	*I*^2^ = 67%	Combined
OS	4	1.40 (1.22, 1.60), *P* < 0.0001	*I*^2^ = 0%	Combined

*OR* odd ratio, *HR* hazard ratio, *CR* complete response, *TR* treatment response, *DC* disease control, *PFS* progression-free survival, *OS* overall survival

Table 4 shows the subgroup analyses based on the different FLTs (CCRT or chemotherapy alone). Based on studies with the first-line CCRT, the combination therapy showed better efficacy in terms of the CR (*P* < 0.0001), TR (*P* = 0.001), DC (*P* = 0.0008) rates, PFS (*P* < 0.00001), and OS (*P* < 0.00001). Based on the studies with chemotherapy alone as the FLT, the combination therapy showed a better efficacy in terms of CR (*P* = 0.0007), TR (*P* < 0.00001), DC (*P* = 0.002) rates, and OS (*P* = 0.0004). The significant heterogeneity was found in the endpoint of PFS (*I*^2^ = 87%).

**Table 4:** Meta-analytic results based on the studies regarding of different first-line treatments

	Number of studies	OR or HR (95% CI)	Heterogeneity	Favor
Concurrent chemoradiotherapy				
CR	4	0.24 (0.13, 0.45), *P* < 0.0001	*I*^2^ = 47%	Combined
TR	4	0.50 (0.33, 0.75), *P* = 0.001	*I*^2^ = 0%	Combined
DC	4	0.31 (0.16, 0.62), *P* = 0.0008	*I*^2^ = 0%	Combined
Myelosuppression	2	0.60 (0.36, 1.03), *P* = 0.06	*I*^2^ = 0%	-
Gastrointestinal response	2	0.91 (0.55, 1.49), *P* = 0.70	*I*^2^ = 0%	-
PFS	4	1.63 (1.32, 2.00), *P* < 0.00001	*I*^2^ = 0%	Combined
OS	3	1.65 (1.33, 2.04), *P* < 0.00001	*I*^2^ = 0%	Combined
Chemotherapy alone				
CR	2	0.22 (0.09, 0.53), *P* = 0.0007	*I*^2^ = 0%	Combined
TR	2	0.11 (0.06, 0.21), *P* < 0.00001	*I*^2^ = 0%	Combined
DC	2	0.08 (0.02, 0.31), *P* = 0.002	*I*^2^ = 46%	Combined
PFS	2	1.41 (0.83, 2.39), *P* = 0.20	*I*^2^ = 87%	–
OS	2	1.34 (1.14, 1.57), *P* = 0.0004	*I*^2^ = 39%	Combined

*OR* odd ratio, *HR* hazard ratio, *CR* complete response, *TR* treatment response, *DC* disease control, *PFS* progression-free survival, *OS* overall survival

## Discussion

In recent years, the treatment for locally advanced, and unresectable NSCLC has been revolutionized by immunotherapy and conventional CCRT [21]. The first-line platinum-based CCRT or chemotherapy is the gold standard for treating resectable, and locally advanced NSCLC [21]. Durvalumab, a PD-L1 suppressor, gained recent FDA approval as a consolidation drug following CCRT; this medication represents a major progress in the treatment of unresectable stage III NSCLC [22]. With consolidation immunotherapy becoming common in managing unresectable stage III NSCLC, the OS has been significantly improved, with the 3-year OS rate reaching up to 57% [23]. Recently, a meta-analysis reported that Endostar could increase the clinical efficacy of CCRT [24].

Approximately 80% of NSCLC patients experienced recurrence within 1–2 years after first-line CCRT [25]. The SLCT is commonly used for treating progressive NSCLC after the FLT [26–28]. However, the TR rates only ranged from 7.7%-18.6%, with the median time to progression of 3.07–3.5 months and the median OS of 7.6–7.83 months [26]. A RCT showed that the 1-year OS rate was 30% after SLCT for progressive NSCLC [28]. These findings indicated that systemic SLCT was not sufficient for treating progressive NSCLC, suggesting that some additional local treatment for the tumor would also required to improve the survival outcome of these patients. Percutaneous CT-guided ISB decreases the local recurrence rate of NSCLC with a few complications [29]. Therefore, many researchers have also combined ISB and first-line chemotherapy for patients with severe NSCLC [7].

In this meta-analysis, we compared the clinical effectiveness of the combination of ISB with SLCT and SLCT alone for patients with progressive NSCLC after they received the FLT. Firstly, the combination therapy promoted a better tumor response rate, which was indicated by the CR, TR, and DC rates. The current results validated the findings from a prior meta-analysis that compared the combination of ISB and chemotherapy with chemotherapy alone as the FLT for severe NSCLC [7]. However, in some of the selected publications in the previously reported meta-analysis, the CR rates in the chemotherapy alone group were less than 5% [12, 16]. These data suggested that ISB could improve the therapeutic efficacy of systemic SLCT by providing the local treatment.

However, the TR (OR = 0.50, *P* = 0.13) and DC (OR = 0.38, *P* = 0.14) rates were comparable between the 2 groups based on the RCT subgroup analysis. This could be because of the limited number of RCTs that were included in this meta-analysis. Furthermore, the 2 included RCTs had only enrolled the patients with stage III NSCLC [11, 14], which might have potentially increased the tumor response rates following the chemotherapy alone regimen.

Our meta-analysis combination group showed a PFS. Furthermore, the combination group showed a longer PFS, and this was true for both the RCTs and retrospective studies that were included in this study. Although, in some cases, SLCT alone might provide similar DC rates when compared to the combination therapy, the combination therapy could effectively prolong the time to tumor progression. OS was also improved, as the PFS was improved by the combination therapy. The effectiveness of the SLCT alone was limited by the dosage-related complications and the individual patients’ tolerance while ISB facilitated the continuous irradiation of the tumors [7].

Myelosuppression and gastrointestinal response are the toxicities associated with chemotherapy. But, we found no obvious differences in myelosuppression and gastrointestinal response between the 2 groups in the present meta-analysis. These findings suggested that ISB did not increase the chemotherapy-related toxic side effects, potentially due to the site-specific nature of ISB therapy.

Apart from the subgroup analysis according to the differing study designs, we also have conducted another subgroup analysis based on the different FLTs (CCRT or chemotherapy alone). We found that both the tumor response rates and the OS were favorable in the combination therapy group, irrespective of the kind of FLT administered. Based on these findings, we concluded that different FLTs did not affect the effectiveness of the combination therapy. Although the PFS duration was similar (HR = 1.41, *P* = 0.20) between the 2 groups based on the subgroup analysis of first-line chemotherapy alone, a significant heterogeneity (*I*^2^ = 87%) indicated that this result required further validation.

This meta-analysis had some limitations. Firstly, a majority of the selected publications were retrospective studies. When we performed the subgroup analysis based on the retrospective studies, many endpoints (TR and PFS) were presented with significant heterogeneity. Further RCTs are therefore required to address this heterogeneity. Secondly, the first-line and SLCT protocols, which included the types of medicines, dosage of the medicines, and the number of cycles of the of treatment, were not same for all the included studies. These parameters may further contribute to the increase in the risk of bias. Thirdly, all the included studies were from China and could contribute to cause the patient selection bias. Therefore, further comprehensive studies involving patients across the globe are required in order to generalize our findings for the wider population.

## Conclusion

In summary, the current meta-analysis demonstrated that ISB could enhance the clinical efficacy of SCLT in patients with progressive NSCLC following FLT without inducing toxic side effects.

## Data Availability

The data that support the findings of this study are available from the corresponding author upon reasonable request.

## Abbreviations

CCRT: Concurrent chemoradiotherapy
CR: Complete response
DC: Disease control
FLT: First-line treatment
ISB: I-125 seeds brachytherapy
NOS: Newcastle–Ottawa scale
NSCLC: Non-small-cell lung cancer
OS: Overall survival
PFS: Progression-free survival
RCT: Randomized controlled trial
SLCT: Second-line chemotherapy
TR: Treatment response
